# CSDPCDA: A miRNA-Mediated Cross-Semantic Regulatory Network Framework for Predicting circRNA–Disease Associations

**DOI:** 10.3390/ijms27188391

**Published:** 2026-09-20

**Authors:** Xin Wang, Mengyuan Zhao, Yixuan Zhao, Sicheng Xiang, Chunyu Wang, Guohua Wang

**Affiliations:** 1 School of Computer Science and Engineering, Northeastern University, Shenyang 110819, China; 2 Key Laboratory of Intelligent Computing in Medical Image, Ministry of Education, Northeastern University, Shenyang 110189, China; 3 Faculty of Computing, Harbin Institute of Technology, Harbin 150001, China

**Keywords:** circular RNA, microRNA, circRNA–disease association, heterogeneous regulatory network, cross-semantic learning, graph attention network

## Abstract

CircRNAs are emerging regulators of human diseases, often functioning through miRNA-mediated regulatory networks. However, experimentally validated circRNA–disease associations remain limited, hindering systematic investigation of circRNA functions and disease mechanisms. Here, we propose CSDPCDA, a miRNA-mediated cross-semantic regulatory network framework for predicting circRNA–disease associations by integrating multi-layer molecular information. CSDPCDA constructs a heterogeneous regulatory network incorporating circRNA–disease associations, circRNA–miRNA interactions, miRNA–disease associations, and molecular similarity information. Multiple biologically meaningful meta-paths are modeled to capture diverse regulatory patterns, and a cross-semantic attention mechanism is employed to integrate complementary molecular representations for association prediction. Comprehensive evaluations on the circR2Disease and circRNADisease datasets showed that CSDPCDA obtained higher AUC, AUPR, and F1-score than the representative existing methods under the same experimental settings. Ablation analyses demonstrated that incorporating miRNA-mediated regulatory information consistently improved the predictive performance across different datasets, while integrating complementary meta-path information further enhanced the characterization of complex molecular regulatory relationships. Moreover, case studies of breast cancer, colorectal cancer, and gastric cancer showed that many of the top-ranked predictions were consistent with previously reported disease-associated circRNAs, providing literature-based evidence for their potential biological relevance. CSDPCDA provides an interpretable framework for prioritizing potential disease-associated circRNAs and facilitating further biological investigation.

## 1. Introduction

Circular RNAs (circRNAs) are a class of endogenous non-coding RNAs characterized by covalently closed loop structures generated through back-splicing events. Initially regarded as transcriptional noise or splicing byproducts, circRNAs have been increasingly recognized as important regulators of gene expression due to their remarkable stability, evolutionary conservation, and tissue-specific expression patterns. Accumulating evidence indicates that circRNAs participate in diverse biological processes through multiple mechanisms, including acting as microRNA (miRNA) sponges, regulating transcription and parental gene expression, and modulating protein interactions [1]. Importantly, aberrant circRNA expression profiles have been extensively observed in various human diseases, particularly cancers, suggesting that circRNAs may serve as potential diagnostic biomarkers and therapeutic targets. Therefore, systematic identification of circRNA–disease associations is essential not only for understanding the regulatory mechanisms underlying disease progression but also for facilitating biomarker discovery and precision medicine.

Despite their biological importance, experimentally identifying circRNA–disease associations remains challenging due to the enormous search space, high experimental costs, and time-consuming validation procedures. Fortunately, the rapid development of high-throughput sequencing technologies and the accumulation of multi-omics biological resources have provided valuable opportunities for computational approaches to uncover potential circRNA–disease relationships. Existing computational methods can be broadly categorized into network-based approaches, traditional machine learning methods, and deep learning-based models.

Early network-based methods mainly constructed heterogeneous biological networks by integrating known circRNA–disease associations with similarity information derived from circRNA and disease characteristics. Subsequently, network propagation or random walk algorithms were employed to infer potential associations based on topological proximity within the network [2]. For example, BRWSP [3], BWHCDA [4], and CD-LNLP [5] effectively utilized network topology information to improve prediction performance. However, these approaches usually rely on manually designed similarity integration strategies, where assigning appropriate weights among heterogeneous biological relationships remains difficult. Moreover, network propagation methods mainly capture local connectivity patterns and may fail to characterize complex nonlinear regulatory mechanisms.

Machine learning-based approaches have further advanced circRNA–disease association prediction by integrating diverse biological features and learning discriminative representations [6,7]. For example, Lei et al. [8] combined random walk-based feature extraction with K-nearest neighbor classification to predict potential associations. Matrix factorization methods, which have been widely applied in recommendation systems, have also demonstrated effectiveness by recovering latent interaction patterns from incomplete association matrices [9,10,11]. Nevertheless, conventional machine learning methods generally depend on handcrafted feature engineering and may have limited capability in capturing high-order nonlinear relationships among heterogeneous biological entities.

With the emergence of deep learning, particularly graph neural networks (GNNs), recent studies have achieved substantial improvements by automatically learning complex representations from biological networks. CNN-based methods have been developed to integrate multi-source biological information [12], while hybrid CNN-RNN frameworks further enhanced feature extraction capability [13]. Autoencoder-based approaches, such as AE-RF and IMS-CDA, have also demonstrated promising performance by learning compact latent representations [14,15]. More recently, GNN-based methods have attracted increasing attention because of their ability to model non-Euclidean biological networks. FastGCN-based models [16], graph attention network approaches [17], and multi-view attention graph convolutional frameworks have successfully exploited graph structural information for circRNA–disease association prediction [18]. Furthermore, Zou et al. [19] designed a method based on the Graph Markov Neural Network algorithm to predict potential circRNA–disease associations. However, most existing GNN-based methods primarily focus on direct circRNA–disease relationships or simple neighborhood aggregation, while overlooking the fact that circRNA functions are often mediated through complex regulatory cascades involving other molecular components.

In particular, circRNAs frequently regulate disease-associated biological processes through interactions with miRNAs, which serve as critical post-transcriptional regulators by controlling target gene expression. Therefore, circRNA–disease associations should not be considered isolated binary relationships but rather consequences of complex multi-layer molecular regulatory networks. Although several existing methods incorporate heterogeneous biological information, they often concatenate different types of similarity features or interaction networks without explicitly modeling the biological semantics underlying different molecular relationships. Furthermore, conventional feature learning strategies usually treat representation learning and association prediction as separate stages, limiting the ability of models to optimize task-specific representations. These limitations highlight the need for computational frameworks capable of integrating multi-level regulatory information while preserving biologically meaningful relational semantics.

To address these challenges, we propose CSDPCDA, a cross-semantic dual-perspective enhancement framework for circRNA–disease association prediction. The proposed method integrates structural modeling and semantic fusion perspectives to comprehensively characterize complex molecular interactions. From the structural perspective, CSDPCDA incorporates miRNA-mediated regulatory information to construct a multi-relational heterogeneous network and employs graph attention-based representation learning along biologically meaningful semantic paths, enabling the extraction of high-order regulatory patterns among circRNAs, miRNAs, and diseases. From the semantic perspective, an adaptive attention mechanism is introduced to dynamically integrate representations from different relational views, thereby generating more informative and discriminative embeddings. These enhanced representations are subsequently incorporated into an inductive matrix completion framework for association prediction. Extensive experiments demonstrate the effectiveness of CSDPCDA, as reflected by its higher predictive performance than the compared methods on benchmark datasets under the same evaluation protocol. Moreover, case studies involving breast cancer, colorectal cancer, and gastric cancer provided literature-supported evidence suggesting that the predicted associations may have potential biological relevance and may represent candidates for further experimental validation.

## 2. Results

### 2.1. Evaluation of Hyperparameters

To investigate the influence of key parameters on the performance of CSDPCDA, four important hyperparameters were evaluated, including the bias parameter (μ), feature representation dimension (dim), number of graph attention layers (Gl), and number of attention heads (K). Hyperparameter tuning and model selection were performed exclusively on the validation set, and model performance was assessed using AUC and AUPR. The independent test set was not involved in hyperparameter optimization and was used only for the final performance evaluation.

**Figure 1:**
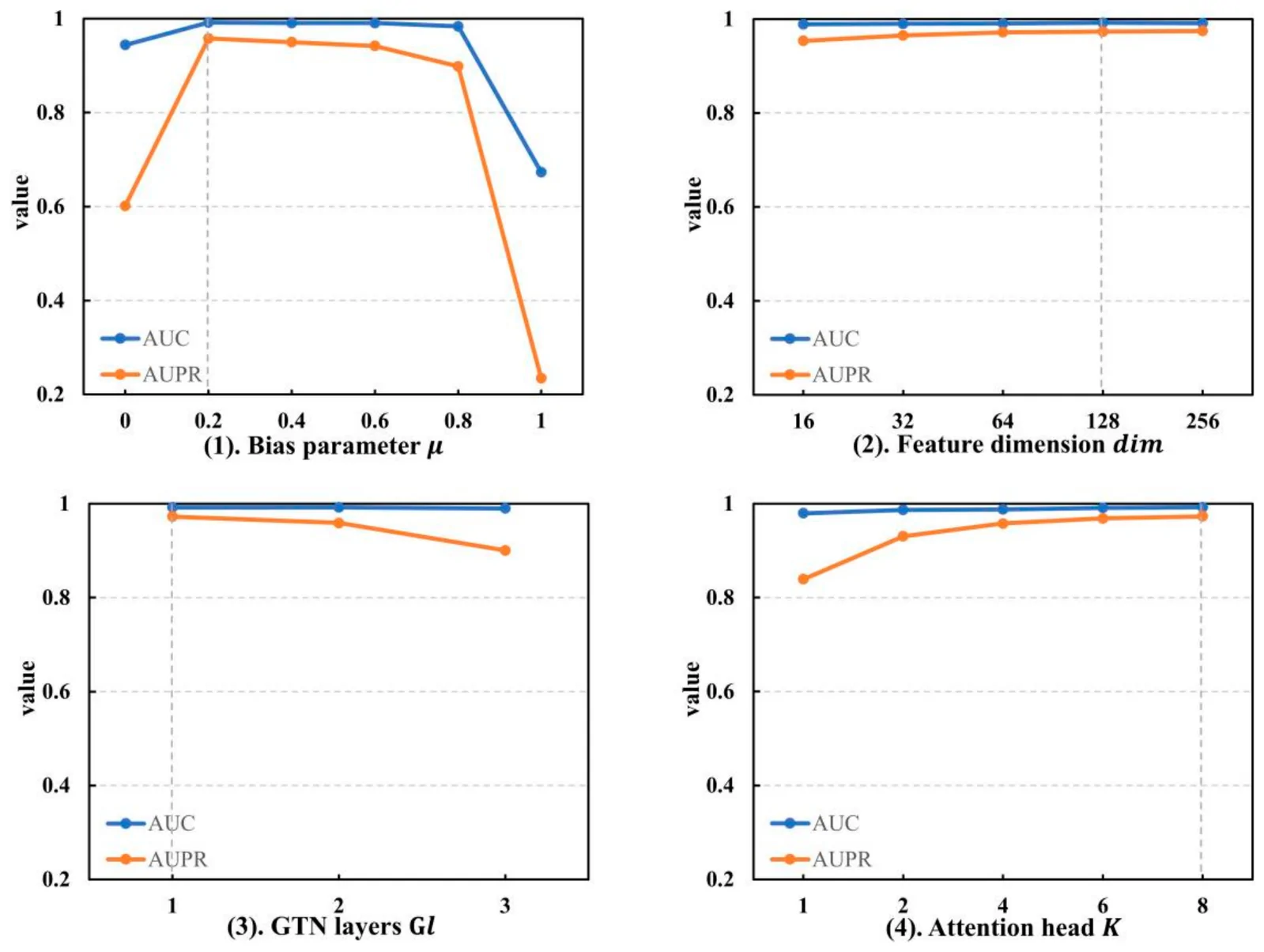
Parameter optimization.

The parameter μ controls the balance between observed associations and unobserved circRNA–disease pairs. As shown in Figure 1(1), the best AUC and AUPR scores were achieved when μ = 0.2, indicating that an appropriate balance between known and unobserved associations contributes to effective association reconstruction.

The feature dimension (dim) determines the capacity of molecular representation learning. As depicted in Figure 1(2), different dimensions were tested, and the best performance was obtained at dim = 128, suggesting that this setting provides sufficient representation capability while avoiding redundant information.

The number of graph attention layers (Gl) affects the extent of information propagation in the heterogeneous network. As shown in Figure 1(3), CSDPCDA achieved the highest performance when Gl = 1, indicating that a shallow graph structure is sufficient for capturing informative molecular relationships.

The number of attention heads (K) was further investigated to evaluate the effect of multi-view feature aggregation. The results in Figure 1(4) show that K = 8 achieved the best performance.

Therefore, the optimal parameter configuration was set as μ = 0.2, dim = 128, Gl = 1, and K = 8, which were selected as the default hyperparameter settings to achieve optimal performance across all evaluation metrics.

### 2.2. Performance Comparison with Existing Methods

To evaluate the predictive capability of CSDPCDA, we compared it with eight representative circRNA–disease association prediction methods, namely DMFCDA [20], CD-LNLP [5], RGCNCDA [21], GCNCDA [16], SIMCCDA [9], CRPGCN [22], AE-RF [14], and GMNN2CD [19]. The AUC and AUPR values were used as evaluation metrics, and all methods were evaluated on the circR2Disease dataset.

**Figure 2:**
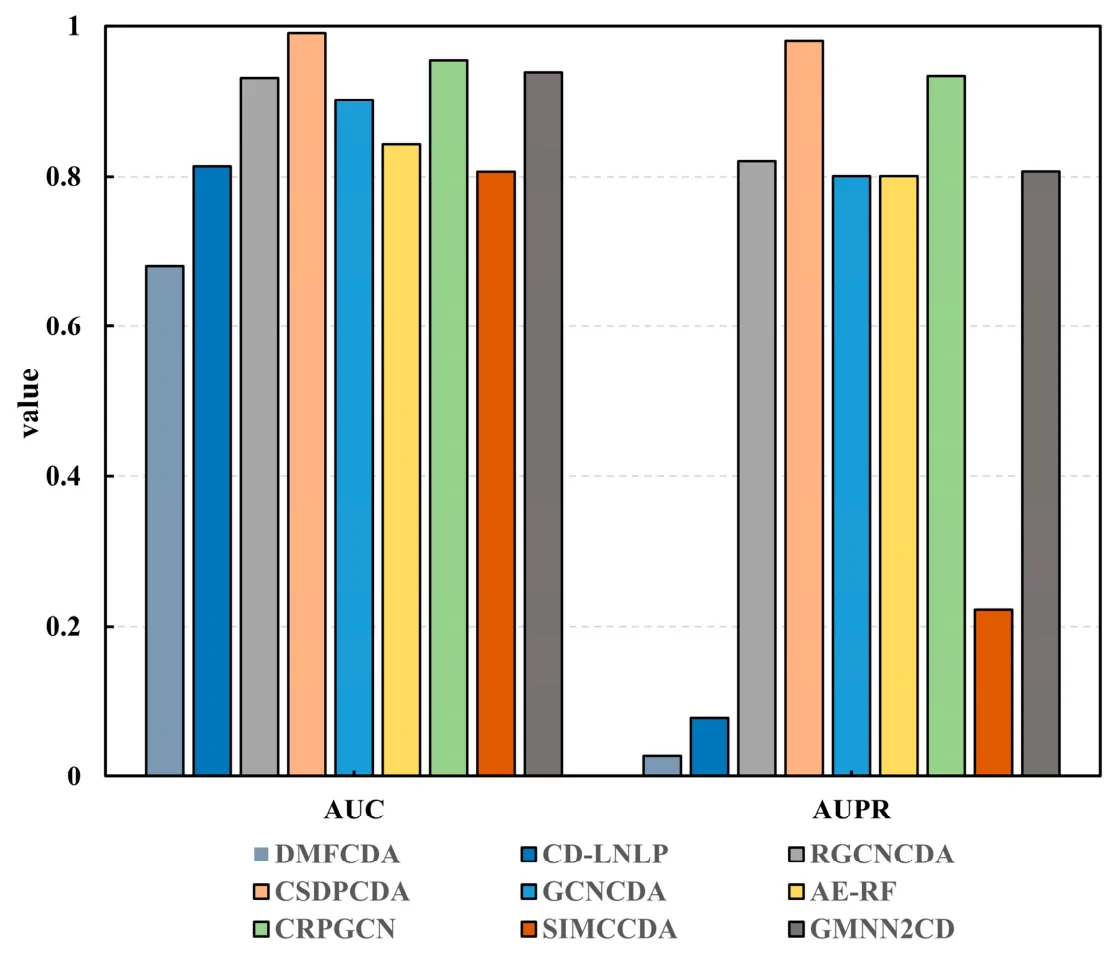
Comparison with other methods on the circR2Disease dataset.

As shown in Figure 2, CSDPCDA achieved the highest AUC and AUPR values among all compared methods, demonstrating its superior capability in identifying potential circRNA–disease associations. Specifically, CSDPCDA improved the AUC value by 3.66% compared with the best-performing baseline CRPGCN and achieved an AUPR of 98.11%, exceeding CRPGCN by 4.74%. These improvements indicate that integrating heterogeneous molecular information through the cross-semantic dual-perspective learning strategy can effectively enhance association prediction performance.

Among the compared approaches, RGCNCDA is a representative method that incorporates miRNA information and utilizes random walk with restart and principal component analysis to obtain molecular representations. Compared with RGCNCDA, CSDPCDA further improved AUC and AUPR by 5.97% and 16.05%, respectively. This improvement can be attributed to the ability of CSDPCDA to simultaneously model local structural dependencies and global semantic relationships within the heterogeneous molecular network. Furthermore, the attention-based semantic fusion mechanism enables adaptive weighting of different regulatory pathways, resulting in more informative molecular representations.

**Figure 3:**
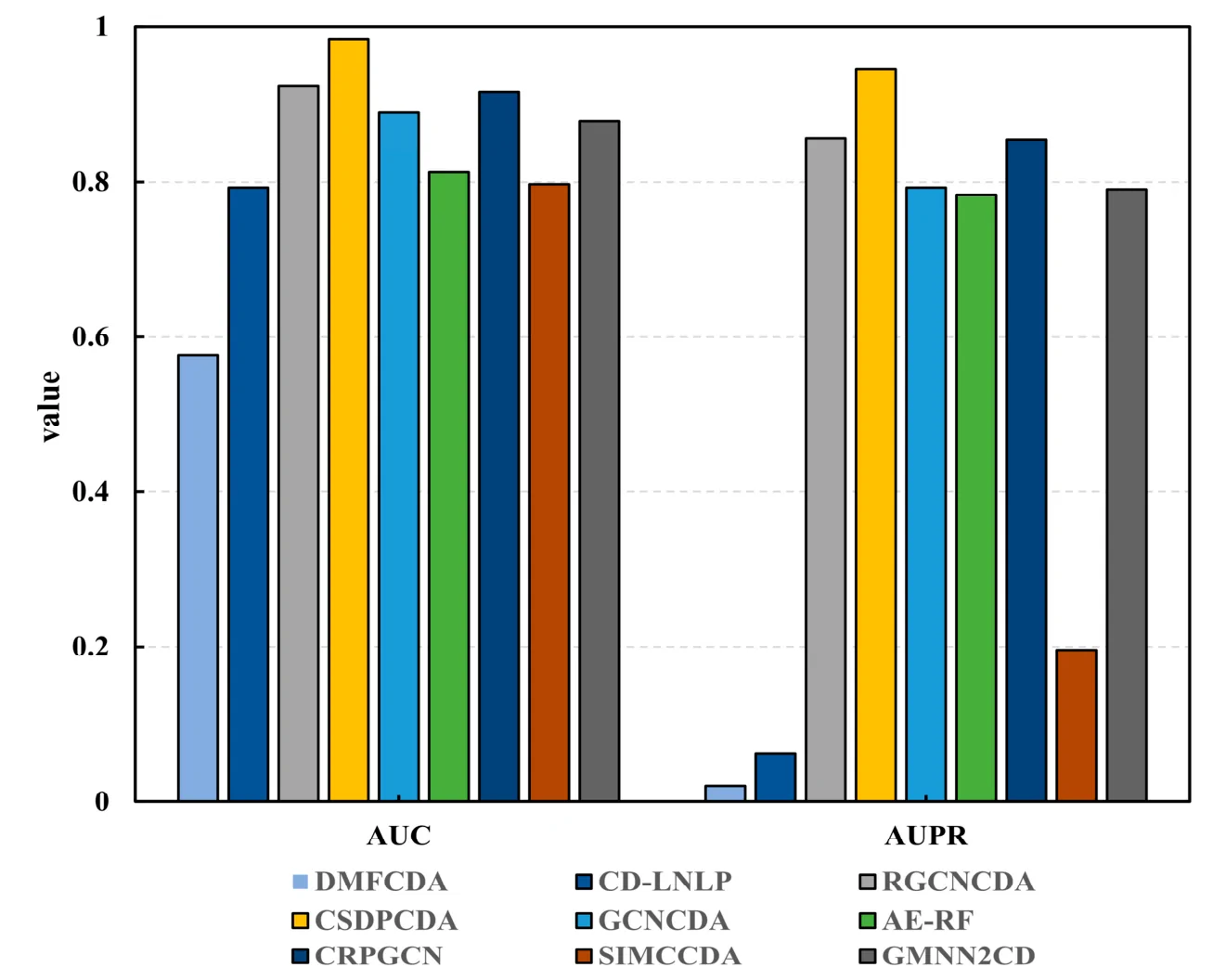
Comparison with other methods on the circRNADisease dataset.

To further assess the generalization capability of CSDPCDA, additional experiments were conducted on the circRNADisease dataset. As shown in Figure 3, CSDPCDA achieved AUC and AUPR values of 98.44% and 94.55%, respectively, representing the highest values among all compared methods under the same experimental setting. Compared with the second-best-performing method, CSDPCDA achieved an AUPR improvement of 8.88%. These results demonstrate that CSDPCDA maintains robust predictive performance across different datasets, highlighting its effectiveness in integrating multi-source biological evidence for circRNA–disease association discovery.

### 2.3. Contribution of miRNA-Mediated Regulatory Information

Since miRNAs serve as important regulatory mediators connecting circRNAs and disease-associated biological processes, we investigated whether incorporating miRNA-mediated information could improve circRNA–disease association prediction. Specifically, both circRNA–miRNA interactions and miRNA–disease associations were integrated into the heterogeneous regulatory network to provide additional molecular context beyond direct circRNA–disease relationships.

**Figure 4:**
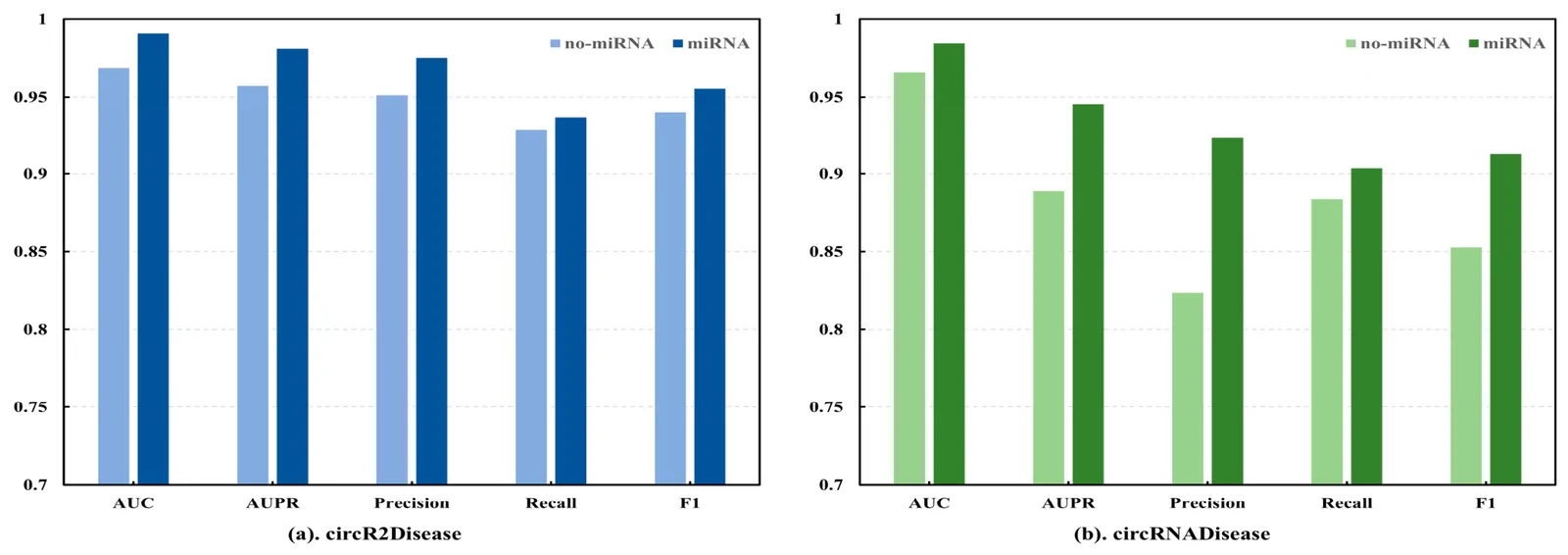
The contribution of miRNA information to model performance.

To evaluate the contribution of miRNA information, two model variants were constructed using the circR2Disease dataset: one incorporating miRNA nodes and their associated regulatory edges (miRNA), and the other removing all miRNA-related information (no-miRNA). As shown in Figure 4a, the model with miRNA information consistently achieved better performance across all evaluation metrics. Compared with the no-miRNA variant, incorporating miRNA information improved the AUC and AUPR values by 2.22% and 2.39%, respectively. The improvement in the F1-score further indicates that miRNA-mediated information contributes to more accurate identification of potential circRNA–disease associations.

To further verify the generalizability of miRNA-mediated information, the same comparison was conducted on the circRNADisease dataset. As shown in Figure 4b, the inclusion of miRNA information resulted in AUC and AUPR improvements of 1.86% and 5.66%, respectively. These results demonstrate that miRNA-associated regulatory information provides complementary biological evidence and enables the model to better capture latent molecular relationships across different datasets.

The improved performance can be attributed to the regulatory bridging role of miRNAs, which connect circRNAs with disease-related biological processes through post-transcriptional regulatory mechanisms. The consistent performance gains after incorporating miRNA-related information indicate that miRNA-mediated regulation provides complementary evidence beyond direct circRNA–disease relationships, rather than simply introducing redundant network information. This finding supports the biological motivation for constructing a miRNA-mediated heterogeneous regulatory network in CSDPCDA.

### 2.4. Effectiveness of Cross-Semantic Regulatory Information Integration

To investigate the contribution of cross-semantic information integration, we evaluated the effectiveness of different meta-path combinations within the heterogeneous regulatory network. Multiple meta-paths were predefined to characterize diverse molecular regulatory contexts among circRNAs, miRNAs, and diseases (Table 1). Since each meta-path captures specific biological relationships, the proposed attention-based fusion strategy was designed to adaptively integrate complementary semantic information and generate more comprehensive molecular representations.

Different combinations of meta-paths were evaluated using AUC, AUPR, precision, recall, and F1-score. As shown in Table 2, incorporating additional complementary semantic paths generally improved prediction performance. Specifically, the complete meta-path fusion strategy achieved the best performance, with an AUPR of 98.11%, a precision of 97.52%, and an F1-score of 95.55%. Compared with partial meta-path combinations, the complete fusion strategy demonstrated substantial improvements, particularly in AUPR and F1-score, indicating enhanced capability in identifying potential positive circRNA–disease associations.

The results demonstrate that individual meta-paths provide complementary biological perspectives, while their integration enables the model to capture more comprehensive regulatory dependencies among molecular entities. Removing a meta-path evaluates whether its corresponding relational context contributes information beyond the remaining paths. The observed performance differences indicate that association and similarity paths capture distinct structural, functional, semantic, or interaction-profile information and are therefore complementary rather than interchangeable. The superior performance of the full semantic fusion strategy further supports the dual-perspective architecture of CSDPCDA, in which relation-specific structural information is first learned from individual meta-paths and subsequently integrated through cross-semantic attention.

### 2.5. Robustness Evaluation Under Independent Negative Sampling

To further assess the robustness of CSDPCDA to negative-sample selection and class imbalance, we performed 10 independent negative samplings on the circR2Disease dataset. In each sampling, 6310 unobserved circRNA–disease pairs were randomly selected as negative samples, together with the 631 known positive associations, resulting in a negative-to-positive ratio of 10:1. Each independently constructed dataset was then evaluated using five-fold cross-validation. Thus, the evaluation consisted of 10 independent sampling experiments, with five-fold cross-validation performed separately within each experiment, resulting in 50 fold-level training-testing runs in total. For each negative sampling, the performance metrics were first averaged across its five cross-validation folds. The final mean ± standard deviation values were then calculated across the 10 independent negative samplings.

As shown in Table 3, CSDPCDA achieved an AUPR of 0.9729 ± 0.0041, an AUC of 0.9920 ± 0.0024, an F1-score of 0.9385 ± 0.0070, a precision of 0.9297 ± 0.0155, and a recall of 0.9483 ± 0.0066. The relatively small standard deviations indicate that model performance remained stable across independently selected negative sets, suggesting that the results were not strongly dependent on a particular negative-sample configuration. Moreover, compared with the original 1:1 balanced benchmark, the AUPR decreased only slightly from 0.9811 to 0.9729 when the negative-to-positive ratio was increased to 10:1. These results provide additional evidence that CSDPCDA maintains stable predictive performance under different negative samplings and a substantially more imbalanced evaluation setting.

### 2.6. Case Study Analysis of Potential Disease-Associated circRNAs

To further evaluate the biological reliability of CSDPCDA in a realistic candidate prioritization setting, we conducted case studies on three representative cancer types, including breast cancer, colorectal cancer, and gastric cancer, using the circR2Disease dataset. For each disease, CSDPCDA was trained using the known circRNA–disease associations, after which unobserved circRNA–disease pairs were scored and ranked according to their predicted association scores. The top 15 candidate circRNAs for each cancer type were selected, and their associations with the corresponding diseases were examined against previously reported evidence in PubMed and other biomedical databases. This evaluation considers the unobserved candidate pairs, thereby providing a large-scale top-K ranking assessment of the model’s ability to prioritize potentially relevant circRNA–disease associations.

As summarized in Table 4, Table 5 and Table 6, a substantial proportion of the top-ranked candidates were supported by previous experimental studies. Specifically, 14 of the top 15 predicted circRNAs for breast cancer, 12 for colorectal cancer, and 12 for gastric cancer were supported by existing literature, corresponding to literature-based validation rates of 93.3%, 80.0%, and 80.0%, respectively. Because the candidates were ranked from the complete set of unobserved pairs for each disease, these results provide an evaluation under a substantially more realistic candidate space than the balanced benchmark. These results suggest that CSDPCDA may be effective in prioritizing biologically relevant circRNA candidates associated with complex diseases for further experimental investigation.

For example, in breast cancer, hsa_circ_001569 has been reported to be upregulated and involved in tumor progression through regulation of the PI3K–AKT signaling pathway [23]. In colorectal cancer, circPVT1 promotes tumor development by acting as a miR-145 sponge [24]. In gastric cancer, circCCDC66 has been associated with cancer cell proliferation, migration, and chemoresistance [25]. These literature-supported cases suggest that some predictions generated by CSDPCDA are consistent with previously reported regulatory mechanisms.

Furthermore, several highly ranked candidates provide additional insights into potential disease-related circRNAs. For instance, hsa_circ_0005986 and ciRS-7 have been implicated in breast cancer regulation through tumor suppressive effects and miRNA-mediated immune regulation, respectively [26,27]. Similarly, hsa_circ_0005927 and hsa_circ_0004771 have shown potential diagnostic relevance in colorectal cancer [28,29], while hsa_circ_0004872 may participate in gastric cancer regulation through negative feedback mechanisms [30]. Collectively, these findings support the effectiveness of CSDPCDA in prioritizing candidate circRNAs and uncovering potential regulatory relationships for further experimental investigation.

## 3. Discussion

Understanding the regulatory mechanisms underlying circRNA–disease associations is essential for elucidating disease progression and identifying potential biomarkers. However, existing computational approaches are often limited by incomplete integration of heterogeneous biological information and insufficient modeling of complex regulatory relationships among different molecular entities. In this study, we developed CSDPCDA, a cross-semantic dual-perspective framework that incorporates miRNA-mediated regulatory information to improve circRNA–disease association prediction.

The major advantage of CSDPCDA lies in its ability to simultaneously capture structural and semantic characteristics within a heterogeneous molecular network. By introducing miRNA-mediated interactions, the model extends conventional circRNA–disease association analysis from direct relationships to multi-layer regulatory relationships involving circRNAs, miRNAs, and diseases. Furthermore, the cross-semantic fusion strategy enables adaptive integration of complementary biological contexts captured by different meta-paths, allowing for a more comprehensive representation of molecular interactions. This design provides a more biologically meaningful framework for uncovering latent circRNA–disease associations compared with approaches relying mainly on direct similarity information or single-type molecular relationships.

Comprehensive evaluations on two benchmark datasets demonstrated that CSDPCDA achieved superior predictive performance compared with existing methods. The consistent improvement obtained after incorporating miRNA-related information highlights the importance of miRNA-mediated regulatory mechanisms in revealing hidden associations between circRNAs and diseases. In addition, the meta-path analysis demonstrated that integrating multiple semantic perspectives contributes to richer molecular representations, suggesting that heterogeneous biological relationships provide complementary information for association discovery.

Beyond computational performance, case studies involving breast cancer, colorectal cancer, and gastric cancer provided literature-supported evidence suggesting the potential biological relevance of the predicted associations. Many highly ranked candidate circRNAs had been reported in previous experimental studies, indicating that CSDPCDA may help prioritize potential disease-related circRNAs for subsequent investigation. These findings suggest that the proposed framework may serve as a useful computational tool for exploring circRNA regulatory functions and generating hypotheses for future experimental validation.

Despite these advantages, several limitations remain. First, the calculation of GIP kernel similarity is based on the known association adjacency matrix. Consequently, the resulting similarity representations may be affected by the sparsity and incompleteness of existing experimentally validated associations, particularly for newly identified circRNAs or diseases with limited known interaction information. Second, due to the lack of experimentally confirmed negative circRNA–disease associations, negative samples were randomly selected from unobserved pairs. Although this widely used strategy was applied consistently across all compared methods, some unobserved pairs may be potential positives, which could introduce false-negative labels and affect performance estimates. Future work will explore high-confidence negative selection and positive-unlabeled learning to mitigate this limitation. Third, although literature-based case studies provide supporting evidence for the potential biological relevance of the predicted associations, further biological experiments and clinical validation are still required before novel predictions can be considered confirmed findings or clinically actionable candidates. In addition, we will develop adjacency-matrix-independent methods to improve model robustness and generalizability to newly discovered circRNAs or diseases.

## 4. Materials and Methods

**Figure 5:**
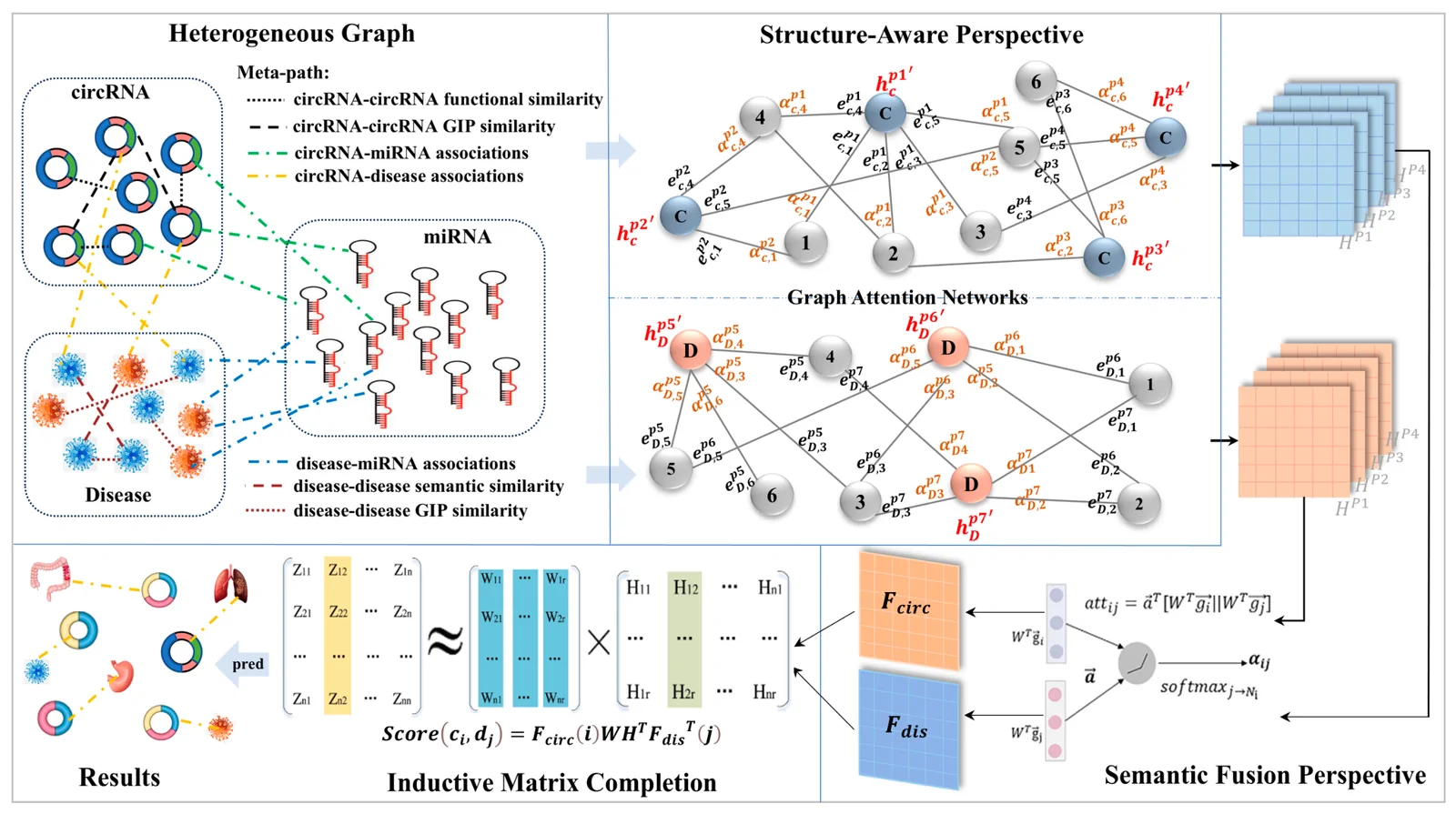
The overall workflow of CSDPCDA.

The overall workflow of CSDPCDA is shown in Figure 5.

### 4.1. Collection and Preprocessing of Dataset

To establish a reliable benchmark for circRNA–disease association prediction, experimentally validated molecular association data were collected from multiple publicly available databases. As shown in Table 7, the collected data included three types of biological relationships, namely circRNA–disease associations, circRNA–miRNA interactions, and miRNA–disease associations, which together provide complementary evidence for modeling circRNA-mediated disease regulation.

The experimentally validated human circRNA–disease association data were obtained from the CircR2Disease database [31]. After removing duplicate entries and standardizing disease names, 631 high-confidence circRNA–disease association pairs were retained, involving 573 circRNAs and 72 diseases. These experimentally confirmed associations were used as positive samples for the circRNA–disease association prediction task.

To incorporate miRNA-mediated regulatory information, miRNA–disease associations were collected from the Human microRNA Disease Database (HMDD) [32]. Following identifier normalization and data preprocessing, 7074 miRNA–disease association pairs were obtained, involving 671 miRNAs and 347 diseases. In addition, circRNA–miRNA interaction data were collected from the circBank database [33], resulting in 7955 circRNA–miRNA association pairs involving 358 circRNAs and 665 miRNAs.

For dataset construction, experimentally validated circRNA–disease associations were used as positive samples. The benchmark dataset contained 631 known associations involving 573 circRNAs and 72 diseases, resulting in 41,256 theoretically possible circRNA–disease pairs. After excluding the 631 known positive associations, the remaining 40,625 unobserved circRNA–disease pairs constituted the candidate pool for negative sampling. Since experimentally validated negative circRNA–disease associations are generally unavailable, we followed previous studies [16] and adopted random sampling to construct the negative sample set. Specifically, 631 non-redundant pairs were randomly selected from the candidate pool and combined with the 631 positive associations to construct a balanced benchmark dataset with a positive-to-negative ratio of 1:1. It should be noted that unobserved circRNA–disease pairs cannot be strictly regarded as experimentally confirmed negative associations, as available biological databases may not yet capture all true associations. Therefore, the sampled pairs were treated as computational negative samples rather than confirmed biological negatives. Although this strategy cannot completely exclude the possibility of false-negative labels, only a small fraction of the substantially larger candidate pool (631/40625≈1.55%) was sampled as negative examples, which helps limit the potential influence of hidden positive associations.

For model evaluation, five-fold cross-validation was performed on the resulting balanced dataset at the association-pair level. In each fold, 80% of the samples were used as the training candidate set, while the remaining 20% were held out exclusively as the test set. The training candidate set was further divided into 90% for model training and 10% for validation. Thus, each cross-validation fold approximately corresponded to a 72%/8%/20% split for training, validation, and testing, respectively. The test samples were used only for final performance evaluation and were not involved in model training or hyperparameter selection.

### 4.2. Heterogeneous Regulatory Network Construction

CircRNAs are increasingly recognized as important regulators of disease-related biological processes through diverse molecular mechanisms, among which miRNA-mediated post-transcriptional regulation represents one of the most extensively studied mechanisms. Therefore, circRNA–disease associations should not be considered isolated binary relationships but rather the consequence of complex interactions among multiple molecular entities. To capture these regulatory dependencies, we constructed a miRNA-mediated multilayer heterogeneous regulatory network by integrating circRNA, miRNA, and disease information from complementary biological perspectives.

The constructed network consists of three types of biological entities, including circRNAs, miRNAs, and diseases. Seven types of relationships were incorporated to characterize different aspects of molecular associations: (i) experimentally validated circRNA–disease associations, (ii) experimentally validated circRNA–miRNA interactions, (iii) experimentally validated miRNA–disease associations, (iv) circRNA functional similarity, (v) circRNA Gaussian interaction profile (GIP) similarity, (vi) disease semantic similarity, and (vii) disease GIP similarity. The experimentally validated associations represent direct molecular relationships, whereas similarity-based relationships provide additional information regarding latent functional and topological similarities among biological entities.

Based on the three experimentally validated association types, three binary adjacency matrices were constructed, namely the circRNA–disease association matrix $A_{\mathrm{CD}},$ the circRNA–miRNA association matrix $A_{\mathrm{CM}}$, and the miRNA–disease association matrix $A_{\mathrm{MD}}$. For each matrix, an element was assigned a value of 1 if the corresponding biological association was experimentally confirmed; otherwise, it was assigned a value of 0. These matrices served as the fundamental biological interaction information for subsequent heterogeneous network construction.

#### 4.2.1. Disease Semantic Similarity

To quantify disease similarity from a biological knowledge perspective, disease semantic information was obtained from the Medical Subject Headings (MeSH) database. Disease-related descriptors belonging to category C were extracted to construct a directed acyclic graph (DAG), where nodes represent disease concepts and edges indicate hierarchical semantic relationships between diseases.

In the constructed DAG, the contribution of disease concept k to disease d, Dd(k), can be calculated as follows:

(1) $$Dd\left(k\right)=\left\{\begin{array}{cc} 1, & if\;k\;=\;d\\ \max\left\{\varphi \;*\;D_{d}(k^{\prime })|k^{\prime }\in children\;of\;k\right\}, & if\;k\;\neq \;d \end{array}\right.$$

where φ represents the semantic contribution factor between a disease concept and its child nodes. The semantic value of disease d, denoted as SV(d), was obtained by summing the contributions of all ancestor concepts:

(2) $$SV\left(d\right)=\sum_{k\in S_{d}}Dd\left(k\right)$$

Based on the assumption that diseases sharing more common ancestor concepts exhibit higher functional similarity [34], the semantic similarity between two diseases d_i_ and d_j_ was calculated as

(3) $$SS\left(d_{i},d_{j}\right)=\frac{\sum_{k\in S_{d\left(i\right)}\cap S_{d\left(j\right)}\;}\left(D_{d_{i}}\left(k\right)+D_{d_{j}}\left(k\right)\right)}{SV\left(d_{i}\;\right)+SV\left(d_{j}\;\;\right)}$$

where k represents the intersection of the ancestor node sets of diseases $d_{i}$ and $d_{j}$, and *Dd_i_*(*k*) represents the semantic contribution value of disease *k* to disease *d_i_*, and *Dd_j_*(*k*) represents the semantic contribution value of disease *k* to disease *d_j_*.

#### 4.2.2. CircRNA Functional Similarity

Since circRNAs involved in similar diseases are likely to participate in related biological processes, circRNA functional similarity was estimated based on the semantic similarity of their associated diseases [35,36,37]. For a disease *d* and a disease set *DS*, the maximum semantic similarity was defined as

(4) $$S\left(d,DS\right)=\begin{array}{c} max\\ 1\leq l\leq L \end{array}\left(SS\left(d,\;ds_{l}\right)\right)$$

For two circRNAs $c_{1}$ and${\;c}_{2}$, their associated disease sets were denoted as DS1 and DS2, respectively. The functional similarity between $c_{1}$ and $c_{2}$ can be defined as

(5) $$S_{\mathrm{Fun}\left(c_{1},c_{2}\right)}=\frac{\sum_{1\leq i\leq M}S\left(\mathrm{ds}1_{i},\mathrm{DS}2\right)+\sum_{\;1\leq j\leq N}\;S\left(\mathrm{ds}2_{j},\mathrm{DS}1\right)}{M+N}$$

where $\mathrm{ds}1_{i}$ represents any disease in the set DS1, and $\mathrm{ds}2_{j}$ represents a disease in the set DS2.

#### 4.2.3. Gaussian Interaction Profile Similarity

To further capture latent similarity patterns from observed biological interaction profiles, Gaussian interaction profile (GIP) kernel similarity was introduced [38,39,40]. GIP similarity assumes that biological entities with similar interaction patterns are more likely to share functional characteristics [41,42,43,44]. Specifically, disease similarity was calculated based on their miRNA association profiles:

(6) $$S_{GIP}=exp\left(-\beta_{d}*\|V\left(d_{i}\right)-V{\left(d_{j}\right)\|}^{2}\right)$$

where $V\left(d_{i}\right)$ and $V\left(d_{j}\right)$ represent binary interaction profiles derived from the miRNA–disease association matrix. $\beta_{d}$ represents the bandwidth parameter of the GIP function, calculated as follows:

(7) $$\beta_{d}={\beta_{d}}^{\prime }\left(\frac{1}{N}\sum_{n=1}^{N}\|V{\left(d_{n}\right)\|}^{2}\right)$$

where N represents the total number of diseases, corresponding to the number of rows in the adjacency matrix *DM*. The GIP similarity of circRNA is also similar.

### 4.3. Dual-Perspective Molecular Representation Learning

Biological regulatory networks usually contain diverse molecular relationships with different biological meanings. Therefore, directly aggregating neighboring information may fail to distinguish the contribution of different regulatory contexts. To address this issue, we designed a dual-perspective representation learning strategy that simultaneously considers structural dependencies and semantic diversity within the heterogeneous molecular network. Specifically, the proposed framework consists of two complementary components: (i) a structure-aware representation module that captures topology-dependent molecular interactions through meta-path-guided graph attention learning and (ii) a semantic fusion module that adaptively integrates representations from different biological relational contexts.

#### 4.3.1. Structure-Aware Representation Learning

In the constructed circRNA–miRNA–disease heterogeneous network, different meta-paths represent distinct molecular regulatory contexts. For example, direct circRNA–disease associations characterize explicit disease-related relationships, whereas circRNA–miRNA–disease paths reflect potential miRNA-mediated regulatory mechanisms. Therefore, we employed graph attention networks (GATs) under different meta-paths to capture path-specific structural dependencies among molecular entities.

For a given meta-path p, the semantic neighbor set of a circRNA node $c_{i}$ is denoted as ${\mathrm{NS}}^{p}\left(c_{i}\right)$, where $c_{j}\in {\mathrm{NS}}^{p}\left(c_{i}\right)$ indicates that $c_{j}$ is connected with $c_{i}$ under the biological context defined by path p. The node representations of $c_{i}$ and $c_{j}$ at the l-th layer are represented as $h_{c_{i}}^{p}$ and $h_{\mathrm{cj}}^{p}$, respectively. To distinguish the importance of different neighboring molecular entities, a path-specific attention mechanism was introduced. The attention coefficient between $c_{i}$ and $c_{j}$ under meta-path p was calculated as

(8) $$e_{ij}^{p}=LeakyReLU({\vec{a}}_{p}^{T}\left[W^{p}h_{c_{i}}^{p}\left|\right|W^{p}h_{c_{j}}^{p}]\right)$$

where a represents a learnable attention vector for meta-path p, enabling the model to quantify the biological relevance between connected molecular entities. W denotes the transformation matrix, and || denotes vector concatenation.

The attention coefficients were normalized using the softmax function:

(9) $$a_{ij}^{p}=softmax\left(e_{ij}^{p}\right)=\frac{exp\left(e_{ij}^{p}\right)}{\sum_{t\in NS^{p}\left(c_{i}\right)}exp\left(e_{it}^{p}\right)}$$

The updated representation of node $c_{i}$ under meta-path p was obtained by aggregating neighbor information weighted by the learned attention coefficients:

(10) $$h_{c_{i}}^{p\prime }=|{|}_{k=1}^{K}\delta \left(\sum_{j\in NS^{p}\left(c_{i}\right)}a_{ij}^{p\_k}\left(W^{p\_k}h_{c_{j}}^{p}\right)\right)$$

where K represents the number of attention heads, and δ denotes a nonlinear activation function. Through this process, the model selectively aggregates informative molecular interactions while preserving the regulatory characteristics associated with different meta-paths. The same strategy was applied to disease nodes to obtain their path-specific representations.

#### 4.3.2. Cross-Semantic Representation Fusion

Although individual meta-paths capture specific molecular relationships, each path only reflects partial biological information. To integrate complementary regulatory perspectives, we introduced a semantic-level attention mechanism to adaptively evaluate the contribution of different meta-path representations.

Assuming that the heterogeneous network contains t semantic paths $\left\{p_{1},p_{2},p_{3},\ldots ,p_{t}\right\}$, for each meta-path $p_{i}$, a semantic importance score was calculated as

(11) $$P_{Score^{pi}}=\frac{1}{n}\sum_{j\in N_{c}}\beta^{T}\sigma \left(Wh_{ci}^{p}+b\right)$$

where $N_{c}$ denote the set of circRNAs, n represents the number of circRNA nodes, W denotes the weight matrix, and β represents the semantic-level attention vector. By normalizing the attention scores, we obtain path-level weights that quantify the contribution of each semantic path embedding to the cross-semantic feature representation:

(12) $$\alpha^{pi}=\frac{\exp\left(P_{Score^{pi}}\right)}{\sum_{i=1}^{t}\exp\left(P_{Score^{pi}}\right)}$$

These weights reflect the relative importance of different biological relational contexts. Finally, the cross-semantic representation of circRNAs was generated by weighted aggregation:

(13) $$F_{circ}=\sum_{i=1}^{t}\alpha^{pi}H^{pi}$$

where $H^{\mathrm{pi}}$ denotes the circRNA representation matrix under meta-path $p_{i}$.

Similarly, the final disease representation matrix $F_{\mathrm{dis}}$ was obtained by applying the same semantic fusion strategy. The resulting circRNA and disease embeddings integrate both structural dependencies and biological semantic information, providing comprehensive molecular representations for subsequent association prediction.

### 4.4. Latent Association Reconstruction via Inductive Matrix Completion

Although a large number of circRNA–disease associations have been experimentally validated, the currently available biological databases still represent only a small fraction of the potential regulatory relationships. The observed associations generally exhibit latent interaction patterns that can be inferred from the intrinsic characteristics of circRNAs and diseases. Therefore, we formulated circRNA–disease association prediction as a latent association reconstruction problem and employed an inductive matrix completion (IMC) framework to infer unobserved molecular associations based on the learned feature representations [45,46,47,48].

Given m circRNAs and n diseases, a circRNA–disease association matrix T was constructed, where each row and column represent a circRNA and a disease, respectively. The element $T\left(i,j\right)$ indicates whether a validated association exists between circRNA i and disease j. Let $\Omega^{+}$ and $\Omega^{-}$ observed positive associations and unobserved associations, respectively. For any entry$\;\left(i,j\right)$, if $\left(i,j\right)\in \Omega^{+}$, then $T\left(i,j\right)=1$; if $\left(i,j\right)\in \Omega^{-}$, then $T\left(i,j\right)=0$.

In this study, the fundamental assumption of IMC is that the association matrix can be generated by applying the feature vectors of row and column entities to a low-rank matrix Z [49]. Based on the observed entries in T, we aim to reconstruct an approximate matrix $Z\in {\mathbb{R}}^{m\times n}$, which can be factorized as $Z={\mathrm{WH}}^{T}$, where $W\in {\mathbb{R}}^{m\times r}$ and $H\in {\mathbb{R}}^{n\times r}$, and r is the minimum rank of W and H. The matrices W and H are obtained by solving the following optimization problem:

(14) $$\begin{array}{l} \begin{array}{c} min\\ W,H,\emptyset_{c},\emptyset_{d} \end{array}\frac{\left(1-\mu \right)}{2}{\left\|Pro_{\Omega^{+}}\left(T-F_{circ}WH^{T}{F_{dis}}^{T}\right)\right\|}_{F}^{2}\\ \;+\frac{\mu }{2}{\left\|Pro_{\Omega^{-}}\left(T-F_{circ}WH^{T}{F_{dis}}^{T}\right)\right\|}_{F}^{2}+\lambda_{1}({\left\|W\right\|}_{F}^{2}+{\left\|H\right\|}_{F}^{2}\\ \;+\lambda_{2}\left(\|\emptyset_{c},{\|}_{F}^{2}+\|\emptyset_{d},{\|}_{F}^{2}\right) \end{array}$$

where $\emptyset_{c}$ and $\emptyset_{d}$ denote the sets of parameters involved in the feature representation learning process for circRNAs and diseases based on the attention mechanism, respectively. μ represents the bias term used to balance known associations and unknown or unobserved associations. $\lambda_{1}$ and $\lambda_{2}$ are regularization parameters that constrain the model using the Frobenius norm (${\left\|.\right\|}_{F}^{2}$) to prevent overfitting. $F_{\mathrm{circ}}$ and $F_{\mathrm{dis}}$ represent the feature matrices of circRNAs and diseases learned through the attention mechanism, respectively.

Finally, the predicted association score between circRNA $c_{i}$ and disease $d_{j}$ is given by

(15) $$Score\left(c_{i},\;d_{j}\right)=F_{circ}\left(i\right)WH^{T}{F_{dis}}^{T}\left(j\right)$$

Higher scores indicate stronger potential associations between circRNAs and diseases.

## 5. Conclusions

In this study, we developed CSDPCDA, a miRNA-mediated heterogeneous molecular network framework for circRNA–disease association prediction and interpretation. By integrating circRNA, miRNA, and disease information and jointly modeling structural and semantic relationships through dual-perspective attention and cross-semantic information fusion, CSDPCDA provides a comprehensive framework for capturing heterogeneous regulatory information underlying circRNA–disease associations. Experimental evaluations, including independent negative-sampling analyses, demonstrated the effectiveness and robustness of the proposed framework under different evaluation settings. Literature-supported case studies further showed its potential for prioritizing biologically plausible candidate associations for subsequent investigation. Overall, CSDPCDA provides a useful computational approach for exploring potential circRNA–disease associations and generating hypotheses for future experimental validation.

## Figures and Tables

**Table 1:** Meta-paths in the circRNA–disease heterogeneous graph.

Meta-Path	Explanation
$p_{1}^{c}$	circRNA–circRNA functional similarity
$p_{2}^{c}$	circRNA–circRNA GIP similarity
$p_{3}^{c}$	circRNA–miRNA associations
$p_{4}^{c}$	circRNA–disease associations
$p_{1}^{d}$	disease–disease semantic similarity
$p_{2}^{d}$	disease–disease GIP similarity
$p_{3}^{d}$	disease–miRNA associations
$p_{4}^{d}$	disease–circRNA associations

**Table 2:** Performance metrics for comparing different meta-path combinations.

Meta-Path Combination	AUPR	Precision	Recall	F1	AUC
$p_{1}^{c}p_{1}^{d}$	0.7026	0.6858	0.6278	0.6496	0.9188
$p_{12}^{c}p_{1}^{d}$	0.7334	0.6986	0.673	0.6776	0.9664
$p_{12}^{c}p_{2}^{d}$	0.7177	0.6984	0.6581	0.6619	0.9418
$p_{12}^{c}p_{12}^{d}$	0.7576	0.7396	0.6349	0.6721	0.967
$p_{123}^{c}p_{12}^{d}$	0.7492	0.7478	0.6546	0.6898	0.968
$p_{123}^{c}p_{13}^{d}$	0.7359	0.7236	0.6271	0.6603	0.9673
$p_{123}^{c}p_{34}^{d}$	0.8008	0.7739	0.7023	0.7303	0.968
$p_{123}^{c}p_{123}^{d}$	0.8178	0.805	0.7521	0.7854	0.9742
$p_{1234}^{c}p_{123}^{d}$	0.9087	0.8991	0.8424	0.869	0.982
$p_{1234}^{c}p_{124}^{d}$	0.9717	0.9279	0.9461	0.9365	0.9922
$p_{1234}^{c}p_{234}^{d}$	0.9716	0.9329	0.9445	0.9383	0.9914
$p_{1234}^{c}p_{1234}^{d}$	**0.9811**	**0.9752**	0.9365	**0.9555**	0.9908

Note: Bold values indicate the best performance achieved by CSDPCDA with the full fusion of all meta-path combinations.

**Table 3:** Performance of CSDPCDA across 10 independent negative samplings with a negative-to-positive ratio of 10:1 on the circR2Disease dataset (mean of 5-fold cross-validation for each sampling).

Sampling	AUPR	AUC	F1	Precision	Recall
1	0.9705	0.9891	0.9435	0.9474	0.9398
2	0.9714	0.9883	0.9393	0.9362	0.9429
3	0.9771	0.9927	0.9407	0.9284	0.9541
4	0.9657	0.9942	0.9277	0.9135	0.9429
5	0.9764	0.9955	0.9428	0.9465	0.9398
6	0.9770	0.9917	0.9390	0.9217	0.9588
7	0.9680	0.9935	0.9248	0.8996	0.9524
8	0.9736	0.9925	0.9444	0.9375	0.9525
9	0.9717	0.9936	0.9454	0.9434	0.9477
10	0.9774	0.9890	0.9372	0.9231	0.9525
Mean±sd	**0.9729 ± 0.0041**	**0.9920 ± 0.0024**	**0.9385 ± 0.0070**	**0.9297 ± 0.0155**	**0.9483 ± 0.0066**

Note: Bold values indicate the mean ± standard deviation of CSDPCDA over 10 independent negative sampling runs for each evaluation metric.

**Table 4:** Prediction of the top 15 circRNAs associated with breast cancer.

Rank	circRNA	Evidence
1	hsa_circ_001569	PMID:31104012
2	hsa_circ_0005986	PMID:35098570
3	hsa_circ_0001417	PMID:32808350
4	hsa_circRNA_100777	PMID:31137165
5	hsa_circ_0006220	PMID:33708563
6	ciRS-7/hsa_circ_0001946	PMID:33544410
7	hsa_circ_0000199	PMID:33495420
8	circPTK2/hsa_circ_0005273	PMID:33436041
9	hsa_circ_0012673	PMID:35794450
10	hsa_circ_0043278	PMID:34800978
11	circUBAP2	PMID:30314706
12	hsa_circ_0001649	Unconfirmed
13	hsa_circ_0001666	PMID: 31187432
14	hsa_circ_0001955	PMID:31187432
15	hsa_circ_0000520	PMID:32922078

**Table 5:** Prediction of the top 15 circRNAs associated with colorectal cancer.

Rank	circRNA	Evidence
1	circPVT1/hsa_circ_0001821	PMID: 30922567
2	circZNF609	PMID: 30570857
3	hsa_circ_101555	PMID:31300733
4	hsa_circ_0000977	PMID:32221048
5	hsa_circ_0005927	PMID: 33312376
6	Circ_HIPK3	PMID:29549306
7	hsa_circ_000605	PMID:30585259
8	hsa_circRNA_0007874/circMTO1	PMID:30556859
9	hsa_circ_0041103	Unconfirmed
10	hsa_circ_0001982	PMID:32141543
11	hsa_circ_0085154	Unconfirmed
12	CircDOCK1	PMID:33685455
13	hsa_circ_0004771	PMID:31737058
14	hsa_circRNA_404833	Unconfirmed
15	circPABPN1	PMID:34453920

**Table 6:** Prediction of the top 15 circRNAs associated with gastric cancer.

Rank	circRNA	Evidence
1	hsa_circ_0001313/circCCDC66	PMID:36698408
2	circRNA_102231	PMID:34374630
3	hsa_circ_0004872	PMID:33172486
4	hsa_circ_0000426	PMID:33996547
5	hsa_circRNA_100782	PMID:32964973
6	hsa_circ_0005603	Unconfirmed
7	circABCB10	PMID:34987010
8	hsa_circRNA_104433	PMID: 33116831
9	circZFR	PMID:29361817
10	circRNA-000911	Unconfirmed
11	circSHKBP1	PMID: 32600329
12	hsa_circ_0001445/circSMARCA5	PMID:28544609
13	CircMAN1A2	PMID:35484118
14	hsa_circRNA_102678	Unconfirmed
15	circRNA_100876	PMID:32305633

**Table 7:** Composition of the dataset.

Relationship	No. of Associations	No. of circRNA/miRNA	No. of Disease/miRNA
circRNA–disease	631	573	72
miRNA–disease	7074	671	347
circRNA–miRNA	7955	358	665

## Data Availability

All datasets used in this study are publicly available and properly cited in the manuscript. CircRNA–disease association data were obtained from CircR2Disease, miRNA–disease association data were retrieved from the Human MicroRNA Disease Database (HMDD), and circRNA–miRNA interaction data were downloaded from circBank. The source code and processed datasets supporting the conclusions of this study are freely available at https://github.com/DeepLabNote/CSDPCDA(accessed on 15 August 2026). The computational framework was implemented using Python (version 3.7, Python Software Foundation, Wilmington, DE, USA), PyTorch (version 1.4.0, Meta, Menlo Park, CA, USA).
